# The Diagnostic Sensitivity of Dengue Rapid Test Assays Is Significantly Enhanced by Using a Combined Antigen and Antibody Testing Approach

**DOI:** 10.1371/journal.pntd.0001199

**Published:** 2011-06-21

**Authors:** Scott R. Fry, Michelle Meyer, Matthew G. Semple, Cameron P. Simmons, Shamala Devi Sekaran, Johnny X. Huang, Catriona McElnea, Chang-Yi Huang, Andrea Valks, Paul R. Young, Matthew A. Cooper

**Affiliations:** 1 Research and Development, Alere, Brisbane, Australia; 2 Oxford University Clinical Research Unit, Hospital for Tropical Diseases, Ho Chi Minh City, Vietnam; 3 Department of Medical Microbiology, Faculty of Medicine, University of Malaya, Kuala Lumpur, Malaysia; 4 Centre for Infectious Disease Research, School of Chemistry and Molecular Biosciences, University of Queensland, Brisbane, Australia; 5 Institute for Molecular Bioscience, University of Queensland, Brisbane, Australia; Tropical Medicine Institute Pedro Kourí (IPK), Cuba

## Abstract

**Background:**

Serological tests for IgM and IgG are routinely used in clinical laboratories for the rapid diagnosis of dengue and can differentiate between primary and secondary infections. Dengue virus non-structural protein 1 (NS1) has been identified as an early marker for acute dengue, and is typically present between days 1–9 post-onset of illness but following seroconversion it can be difficult to detect in serum.

**Aims:**

To evaluate the performance of a newly developed Panbio® Dengue Early Rapid test for NS1 and determine if it can improve diagnostic sensitivity when used in combination with a commercial IgM/IgG rapid test.

**Methodology:**

The clinical performance of the Dengue Early Rapid was evaluated in a retrospective study in Vietnam with 198 acute laboratory-confirmed positive and 100 negative samples. The performance of the Dengue Early Rapid in combination with the IgM/IgG Rapid test was also evaluated in Malaysia with 263 laboratory-confirmed positive and 30 negative samples.

**Key Results:**

In Vietnam the sensitivity and specificity of the test was 69.2% (95% CI: 62.8% to 75.6%) and 96% (95% CI: 92.2% to 99.8) respectively. In Malaysia the performance was similar with 68.9% sensitivity (95% CI: 61.8% to 76.1%) and 96.7% specificity (95% CI: 82.8% to 99.9%) compared to RT-PCR. Importantly, when the Dengue Early Rapid test was used in combination with the IgM/IgG test the sensitivity increased to 93.0%. When the two tests were compared at each day post-onset of illness there was clear differentiation between the antigen and antibody markers.

**Conclusions:**

This study highlights that using dengue NS1 antigen detection in combination with anti-glycoprotein E IgM and IgG serology can significantly increase the sensitivity of acute dengue diagnosis and extends the possible window of detection to include very early acute samples and enhances the clinical utility of rapid immunochromatographic testing for dengue.

## Author Summary

Dengue is a serious public health concern with around 3 billion people at risk of infection. Severe forms of the infection can be fatal and with no licensed vaccine or effective therapeutic currently available, early detection is important to assist with the clinical management of symptoms. Isolation of the virus and the detection of viral RNA using RT-PCR are commonly used methods for early diagnosis but are time-consuming, expensive and require skilled operation. Rapid immunochromatographic tests (ICT) are relatively simple, inexpensive and easy to perform at or near the point of care. Here, we report on the clinical performance of a new rapid ICT for the non-structural protein 1 (NS1) of dengue virus, a marker of acute infection. At two clinical study sites, NS1 was detected in 60–70% of laboratory-confirmed dengue cases and specificity of the test was >95%. We have also shown that a combined testing approach for both circulating NS1 antigen and antibody responses to the glycoprotein E of the virus can significantly improve diagnostic sensitivity compared to the detection of NS1 alone. Importantly, the combined antigen and antibody testing approach also provides an expanded window of detection from as early as day 1 post-onset of illness.

## Introduction

Dengue is a significant public health threat, with estimates of 50 to 100 million cases per year and around 3 billion people at risk of infection [1]. There have now been epidemics reported in over 100 countries and they appear to be occurring more frequently [2]. The global burden from dengue infections is likely to be much higher than current prevalence data suggests with only 10% of symptomatic cases believed to be reported [3]. Dengue is a febrile illness caused by the dengue virus, a group of single stranded RNA viruses belonging to the *Flaviviridae* family and *Flavivirus* genus. The four serotypes (DENV1-4) of dengue virus are transmitted to humans primarily via *Aedes aegypti* mosquitoes. Infection can result in a broad spectrum of disease syndromes ranging from an asymptomatic or mild infection, classical dengue fever (DF), to the potentially fatal dengue haemorrhagic fever (DHF) and dengue shock syndrome (DSS) [4]. Classical DF is characterised by a sudden onset of fever with a combination of severe headache, retro-orbital pain, myalgia, arthralgia, rash, nausea and vomiting. DHF and DSS are also characterised by increased vascular permeability, thrombocytopenia and haemorrhaging with haemoconcentration being a key indicator in differentiating it from DF [5].

A primary infection confers lifelong protective immunity against the infecting serotype but not cross-protection against any of the other three serotypes [6]. A risk factor for DHF and DSS is a secondary infection with a heterologous serotype. Other risk factors include age, duration between dengue infections, ethnicity as well as the serotype and genotype of the infecting virus [7].

There is currently no licensed dengue vaccine available and the only means of prevention is through surveillance and vector control. There is also no effective anti-viral therapeutic on the market and supportive therapy such as fluid replacement is the only treatment for severe forms of the disease. An early and accurate laboratory diagnosis of dengue could assist clinical management. Virus isolation coupled with immunofluorescence is an effective technique for early diagnosis of dengue. However, it is time-consuming and due to the short duration of viraemia is only sensitive for samples taken up to 5 days post-onset of infection [8]. Real time RT-PCR has also proven to be a highly sensitive and powerful alternative to virus isolation as it has a wider window of detection and allows for both quantitation of viral loads and serotyping [8]. However, molecular techniques such as PCR require specialised equipment, and there has been little progress in the standardisation of protocols, which has limited their utility in lower socio-economic countries where there is a need for simple and affordable testing.

Relatively inexpensive serological tests, and in particular rapid point-of-care devices, have become more widely used in endemic settings. Primary dengue infection is characterised by the presence of significant or rising levels of IgM 3–5 days after the onset of infection, which can persist for 1–3 months, whereas secondary infection is characterised by elevated IgG levels, reaching a peak window of detection at 6–15 days after onset of symptoms and may also be accompanied by IgM [5], [9]. The Dengue Duo Cassette is designed to detect IgM antibodies to dengue, as well as elevated IgG titers that are indicative of a secondary infection. The assay is intentionally designed to not detect low level IgG antibodies from past exposure, which is common in individuals from endemic regions. In the last decade, dengue non-structural protein 1 (NS1) has been identified as a useful early serum marker for primary and secondary dengue infections, and is typically present between days 1–9 after onset of clinical signs, with a peak from days 3–5 [1], [2], [10]–[13]. The Dengue Early Rapid is a 15-minute immunochromatographic test (ICT) designed for the qualitative detection of dengue NS1 antigen in human serum. Although not as sensitive as PCR or ELISA, ICTs are quick and require only a minimum of technical expertise to perform. Here, we report on the clinical performance of a new commercial dengue NS1 detection test, evaluated both alone and in combination with a dengue IgM and IgG rapid test, at two separate study sites in Southeast Asia.

## Methods

### Affinity and specificity of anti-NS1 monoclonal antibodies used in assay development

Anti-Dengue NS1 monoclonal antibodies (Ab1, Ab2 and Ab3) were generated as part of a previous ARC Linkage grant between Alere, Australia and the University of Queensland (ARC LP0668437). Interaction specificities and affinities were determined using a surface plasmon resonance (SPR) biosensor. The SPR assay also allowed for the determination of interaction kinetics. All reagents and sensor chips were purchased from GE Healthcare unless otherwise indicated. Recombinant DENV1, DENV3 and DENV4 NS1 were provided by Alere Inc. (Biosite, U.S.A). Recombinant DENV2 NS1 was supplied by Hawaii Biotech (U.S.A).

SPR experiments were run on a Biacore T100 instrument (GE). Binding kinetics of anti-Dengue NS1 antibodies were measured at 25°C using a CM5 sensor chip. An antibody-based capture method [14] was used to capture the anti-Dengue NS1 mAbs. In brief, an anti-mouse capture kit (BR-1008-38) and an amine coupling kit (BR-1000-50) were used to immobilize approximately 12,000 RU of anti-mouse IgG. Following immobilization, 5 µL of 10 nM anti-Dengue NS1 mAbs were injected over flow cell 2 (Fc2) at a flow rate of 5 µL/min (leading to 50–70 RU of immobilised material), with flow cell 1 (Fc1) used as an un-derivatised reference. In order to investigate the binding kinetics of anti-NS1 mAbs, a kinetic titration (single cycle kinetics) method was utilised [15]. This method involved injecting an analyte concentration series in one single cycle without regeneration steps. Dengue NS1 antigens were prepared in a 3-fold dilution series in HBS-EP running buffer (10 mM HEPES pH 7.4, 300 mM NaCl, 3 mM EDTA and 0.005% Surfactant P20) at a concentration range of 1000 nM to 12.3 nM. The concentration series of each antigen was then injected sequentially in order of increasing concentration over both Fc1 and Fc2 at a flow rate of 30 µL/min for 3 min within one single cycle, with a dissociation phase monitored for 30 min. The sensor surface was regenerated between cycles with 10 mM glycine-HCl pH 1.7 for 40 seconds at a flow rate of 30 µL/min. Each analysis was repeated at least three times. Mass transport limitation of binding was determined to be negligible as the observed binding rate (*k_obs_*) of antigen at a range of flow rates was found to vary by less than 5%.

Data analysis was carried out using Biacore T100 Evaluation software 2.0 (GE). The association rate constants (*k_a_*) and dissociation rate constants (*k_d_*) were determined by the evaluation of the single cycle kinetc sensorgrams, and the equilibrium disassociation constants (*K_D_*) were derived from the ratio *k_d_*/*k_a_*. During the kinetic analysis, a 1∶1 binding model was applied preferentially. This model was sufficient to accurately describe the binding data when an extremely low level of captured antibody was used. When higher levels of antibody were captured, bivalent (second order) binding kinetics was observed, and a bivalent analyte binding analysis model was applied to better fit the kinetic parameters (data not shown).

### Analytical sensitivity of Dengue Early Rapid antigen test

The theoretical limits of detection for each dengue NS1 serotype were determined using both recombinant and native antigen preparations. A serial two-fold dilution series of each recombinant NS1 serotype was prepared in Serasub® with 5% protease-free BSA fraction V, 0.1% sodium azide, 50 µg/mL gentamicin (SBAG) from 512 ng/mL to 0.5 ng/mL, in triplicate. For native NS1, culture supernatants from Vero cells infected with DENV serotype 1 (East Timor isolate, Genbank ID ET00243), serotype 2 (New Guinea C isolate 1944), serotype 3 (East Timor isolate, Genbank ID ET00209) or serotype 4 (East Timor isolate, Genbank ID ET288) were harvested at days 3–5 or 7–10 post-infection, depending on the multiplicity of infection and serotype. The concentration of NS1 in the supernatants was estimated using a quantitative ELISA [13]. A serial two-fold dilution of supernatants was prepared in SBAG. SBAG alone was included as a blank. Assays were performed as per the instructions for use. Briefly, 50 µL of sample was added to a tube followed by 25 µL of Running Buffer and 25 µL of colloidal gold conjugated to an anti-NS1 monoclonal antibody. After mixing, a test strip was added to the tube and incubated for 15 min at RT. The absorbance of the test line was measured using a Hamamatsu MS1000 strip reader. Absorbance data was plotted and analysed using GraphPad Prism 4 software version 4.03. The limit of detection for each serotype was determined as the concentration required to produce an absorbance value of 8.5 mABS units (cut-off for visualisation of test line) by interpolation from a polynomial non-linear regression.

### Clinical evaluation sites and samples

The Dengue Early Rapid test was independently evaluated alone or in combination with the Dengue Duo Cassette in retrospective studies at two Southeast Asian sites. Commercial tests were provided by Alere, Australia and testing was performed as outlined in the instructions for use of the test. For both sites the operators involved in the testing were experienced laboratory technicians working in ISO15189 accredited diagnostic laboratories. All operators received formal training in the methods and in their interpretation and were blinded to the final reference diagnosis.

An evaluation of the Dengue Early Rapid was conducted at the Oxford University Clinical Research Unit in Vietnam using stored acute plasma samples from 100 clinically suspected dengue patients but with no laboratory evidence of acute or recent dengue virus infection and 198 samples from patients with laboratory-confirmed dengue. Samples were collected as part of a prospective hospital-based study of early dengue in Dong Thap Hospital, Dong Thap, Vietnam. The median age was 10 yrs (range 3–15 yrs) and all patients provided written informed consent to participate in the study. The study was approved by the Dong Thap Hospital Ethical Committee and the Oxford Tropical Research Ethical Committee. Plasma samples used in the Dengue Early Rapid evaluation were collected from patients 1–4 days post-onset of dengue-like symptoms. Laboratory-confirmed cases were defined by the detection of DENV RNA in plasma by RT-PCR [16] or seroconversion in the IgM or IgG antigen-capture ELISA [17] in a patient for whom there was a clinical suspicion of dengue (see Figure S1A for the testing algorithm used at Vietnam study site). A “not dengue” case was defined by the absence of virological or serological evidence of DENV infection in a patient where two paired plasma specimens were available with the 2nd specimen taken a minimum of 2 days after the first and at least 5 days after the onset of illness (Figure S1A). Reference testing was performed 6 months prior to the evaluation of the rapid test. The vast majority of the samples from this panel were collected on day 3 post-onset of illness (59.4%), with 13.8% collected on day 2, 12.1% on day 4 and only 2.7% from day 1. In 12% of the samples information on the day of collection was unavailable (Table 1).

**Table 1:** Human serum panel in Vietnamese study site (n = 298).

Days post-onset of illness	RT-PCR Serotyping				Serology‡	Negative	Total
	DENV-1	DENV-2	DENV-3	DENV-4			
1	1	0	1	0	2	4	8
2	9	4	5	0	4	19	41
3	60†	14	20	2	22	59	177
4	13	6	3	1	8	5	36
UNKNOWN*	18	0	2	0	3	13	36
Total	101	24	31	3	39	100	298

†One sample was positive for both DENV-1 and DENV-2 by RT-PCR

‡Negative by RT-PCR and Positive by IgG and IgM ELISA

*No information was available for day post-onset of illness that specimen was taken.

A second retrospective study of the Dengue Early Rapid used in combination with the Dengue Duo Cassette was performed at the University in Malaya. Positive specimens were obtained from a dengue surveillance study conducted at the University of Malaya Medical Centre (UMMC) from 2005 to 2008 and were selected to represent a broad spectrum of patient presentation from 1–15 days post-onset of illness. The study was approved the UMMC Scientific and Ethics Committee. The median age of the patients from this study was 25 yrs (range 1–67 yrs). The serum panel included 263 samples laboratory-confirmed as positive for dengue infection and 30 dengue negative samples from patients diagnosed with an alternative illness, including Epstein-Barr Virus (n = 10), Cytomegalovirus (n = 10) and BK virus (n = 10) aetiologies. Specimens were collected and tested for dengue using a combination of virus isolation [18], haemagglutination inhibition [19], RT-PCR [20], IgM capture ELISA [21], IgG capture ELISA [22] and NS1 Capture ELISA [23] and were considered positive if confirmed by two or more testing methods (see Figure S1B for the testing algorithm used at Malaysia study site). Reference testing was performed immediately prior to the evaluation of the two rapid tests. Negative cases were collected from a routine diagnostic service provided by the University of Malaya and were confirmed as dengue negative by the absence of antibodies and NS1 (Figure S1B). No information on patient age was available from the negative collection.

### Statistical analysis

All statistical analysis was performed using SPSS version 12.0.1. Clinical data was expressed with 95% confidence intervals. Clinical sensitivity between groups were compared by Fisher's exact test (two-sided) and was considered significant if P<0.05.

## Results

### Binding kinetics and specificity of anti-NS1 monoclonal antibodies used for antigen capture

The binding affinities of anti-NS1 antibodies (Ab1, Ab2 and Ab3) used in the Dengue Early Rapid were determined by SPR. Anti-NS1 antibodies were bound to a SPR sensor surface using the capture method as described in the Methods section. The Ab2 antibody specifically bound to DENV1 and DENV2 NS1 with no significant binding observed for DENV3 or DENV4. The Ab1 MAb effectively bound three of the four serotypes of NS1 with no detectable binding to DENV1. Lastly, the Ab3 MAb was observed to cross-react with all four NS1 serotypes.

**Figure 1:**
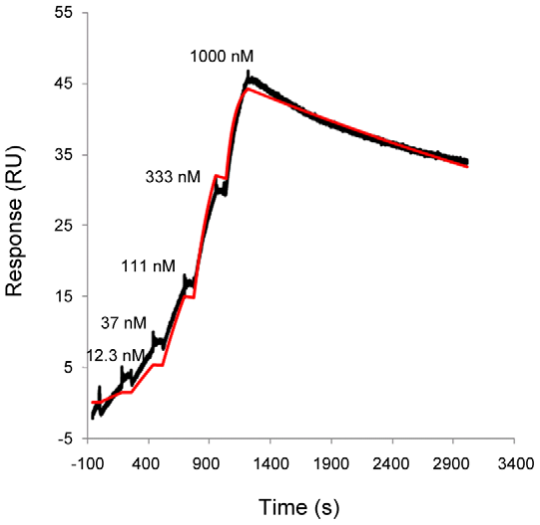
Representative sensorgrams for binding of monoclonal antibody Ab3 and DENV-1 NS1. Increasing concentrations of dengue-1 NS1 were sequentially injected onto 70 RU of Ab3 captured by anti-mouse IgG. The reference-subtracted data (black curve) were fitted using 1∶1 binding algorithm (red curve).

In order to promote monovalent binding interactions that facilitated facile determination of affinity and Langmuir binding (analysis of binding kinetics), we decreased the capture level of anti-NS1 antibodies to 50–70 RU. As a result, acceptable fitting of the data could be obtained by the monovalent binding algorithm (Figure 1). The dissociation rate constants *k_a_*, *k_d_* and *K_D_* derived from a 1∶1 binding model are reported in Table 2. The analysis revealed that the *k_a_* values of the three MAbs were similar for all recognised Dengue NS1 antigens, with the exception of Ab2 for DENV1, which had a 2–3 fold faster on-rate than the others. We observed that the *k_d_* values of the three MAbs were also comparable with the exception of Ab1 for DENV3, which had a faster dissociation rate than the others.

**Table 2:** Binding kinetics of anti-NS1 MAbs to dengue NS1 antigens determined by 1∶1 binding model.

Anti-NS1 mAb	NS1 antigen	*k_a_* [M^−1^s^−1^] ×10^4^	*k_d_* [s^−1^] ×10^−4^	*K_D_* [nM]
**Ab1**	DENV1 NS1	N/A	N/A	N/A
	DENV2 NS1	2.1±0.04	2.4±0.06	11.5±0.5
	DENV3 NS1	1.6±0.20	14.8±4.9	96.5±4.3
	DENV4 NS1	2.1±0.02	1.1±0.05	51.0±2.1
**Ab2**	DENV1 NS1	4.8±0.05	2.59±0.04	5.4±0.1
	DENV2 NS1	2.8±0.06	3.54±0.03	12.3±0.2
	DENV3 NS1	N/A	N/A	N/A
	DENV4 NS1	N/A	N/A	N/A
**Ab3**	DENV1 NS1	1.4±0.03	1.6±0.03	11.9±0.5
	DENV2 NS1	1.9±0.01	1.3±0.04	6.9±0.2
	DENV3 NS1	1.1±0.02	4.7±0.02	41.6±1.1
	DENV4 NS1	1.2±0.00	3.7±0.07	31.2±0.6

*K_D_* was calculated by *k_d_*/*k_a_*. Values are mean ± standard deviation from at least three experiments.

On the basis of this specificity data the Dengue Early Rapid test was developed using an equimolar combination of Ab2 and Ab1 MAbs immobilised onto nitrocellulose membrane with the cross-reactive Ab3 MAb conjugated to colloidal gold particles for detection of captured NS1. The ability of the test to detect all four serotypes of NS1 was verified by assessing the theoretical limits of detection.

### Limit of detection for each dengue NS1 serotype

**Figure 2:**
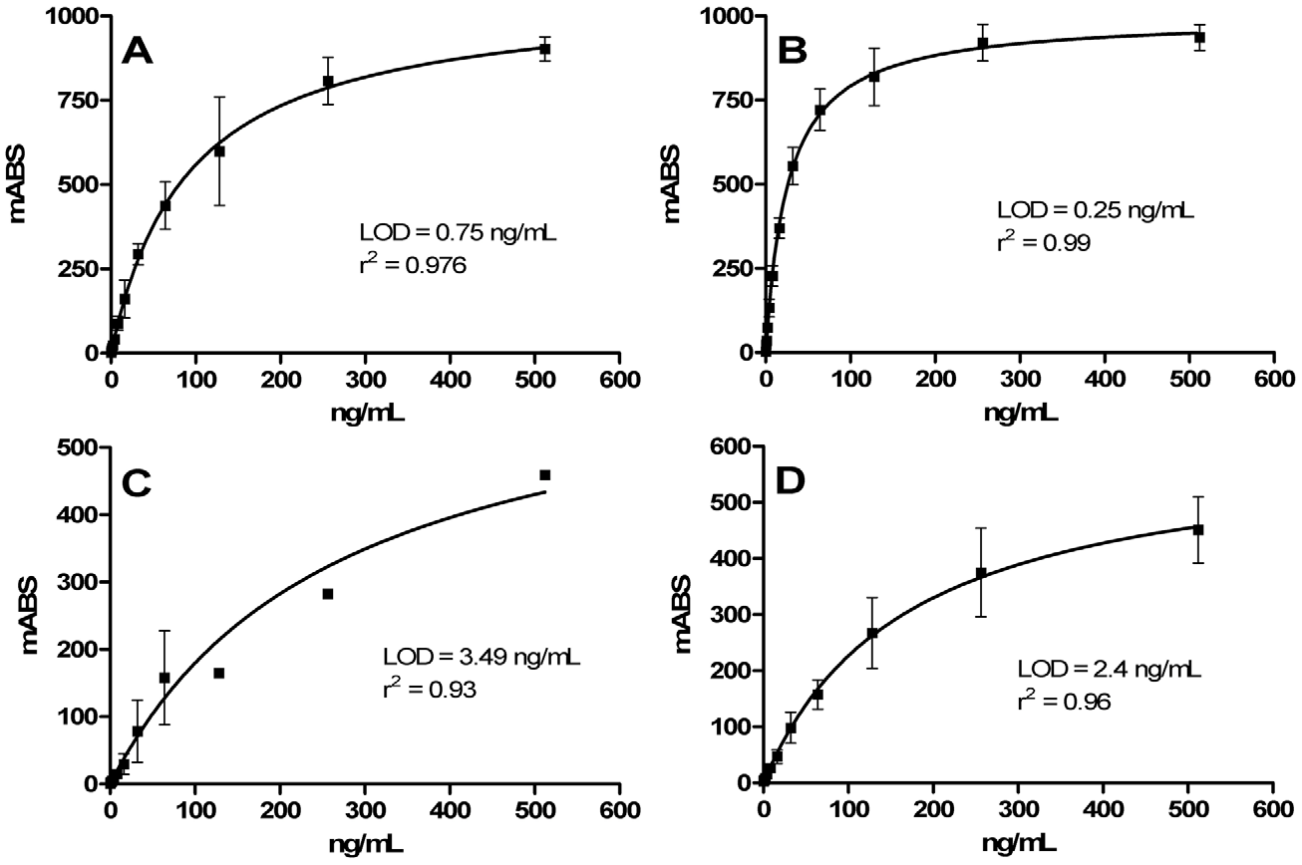
Analytical sensitivity of Dengue Early Rapid test for recombinant dengue NS1. The test line signal intensity was measured against a serial two-fold dilution series of DENV-1 NS1 (A), DENV-2 NS1 (B), DENV-3 NS1 (C) and DENV-4 NS1 (D) from 512 ng/mL to 0.5 ng/mL. The analytical limit of detection was determined as the lowest concentration required to produce a positive result of 8.5 mABS (absorbance) units using interpolation from a non-linear regression. An absorbance value of 8.5 mABS was determined to be the cut-off value for visualisation of the test line.

The analytical sensitivity of the Dengue Early Rapid was determined using recombinant NS1 of each four serotypes to be <5 ng/mL, with a limit of detection of 0.75 ng/mL for DENV-1, 0.25 ng/mL for DENV-2, 3.5 ng/mL for DENV-3 and 2.4 ng/mL for DENV-4 NS1 (Figure 2). The limit of detection for NS1 secreted from dengue-infected Vero cells was comparable to the recombinant proteins with 4.4 ng/mL for DENV-1, 1.4 ng/mL for DENV-2, 5.2 ng/mL for DENV-3 and 1.5 ng/mL for DENV-4.

### Clinical performance of Dengue Early Rapid test at Vietnam study site

When compared to the 198 laboratory-confirmed dengue cases, as indicated by the detection of viral RNA by RT-PCR and/or seroconversion by IgM and IgG capture ELISA, the Dengue Early Rapid had a sensitivity of 69.2% (95% CI: 62.8–75.6) (Table 3). In the 159 of 198 samples that were positive by RT-PCR, the sensitivity of the rapid test was marginally higher with NS1 detected in 71.1% of cases (95% CI: 64.0–78.1). Conversely, in samples that demonstrated seroconversion with no viral RNA detected (39 of 198) the sensitivity of the rapid test was lower, with NS1 detected in 61.5% (95% CI: 46.2–76.8). The specificity of the rapid test was 96% (95% CI: 92.2–99.8), with four false positives observed from the panel of 100 endemic non-dengue specimens (Table 3). When the performance of the NS1 rapid test format was directly compared to an NS1 capture ELISA (BioRad Platelia), the rapid was found to have a lower sensitivity, with 69.2% versus 83.8% respectively, but showed equivalent specificity.

**Table 3:** Accuracy of Dengue Early Rapid (NS1) test in Vietnamese study site.

Samples	n	Dengue Early Rapid		
		agreement	%	95% CI
Positive	198	137	69.2	62.8 – 75.6
Negative	100	96	96.0	92.2 – 99.8

Although all four dengue virus serotypes were represented in the population of positive samples, DENV-1 was the predominant serotype with 62.8% of PCR-confirmed samples with fewer DENV-3 (19.5%), DENV-2 (15.9%) and DENV-4 (1.9%) cases (Table 1). The sensitivity of the antigen rapid test for each serotype was 78% for DENV-1, 62.5% for DENV-2, 61.3% for DENV-3 and 33.3% (1/3) for DENV-4.

### Clinical performance of a combined antigen and antibody testing protocol at Malaysia study site

At the Malaysia study site the predominant serotype was also DENV-1 with 62.7% of PCR-confirmed samples with 14.3% DENV-3, 13% DENV-2 and less than 10% DENV-4 (Table 4). Of the 161 PCR positive samples tested at the Malaysia site, 111 produced a positive result on the Dengue Early Rapid for a sensitivity of 68.9% (95% CI: 61.8 – 76.1). The relative sensitivity of the NS1 rapid test for each serotype was 69.3% for DENV-1, 62% for DENV-2, 78.3% for DENV-3 and 62.5% for DENV-4. NS1 antigen was also detected in 51% of positive samples that were RT-PCR negative but positive by serology. The sensitivity of the NS1 rapid was found to be slightly lower than an NS1 capture ELISA (Panbio® Dengue Early ELISA). The ELISA had a sensitivity of 68.4% against confirmed acute dengue samples compared to 62% for the rapid test. This was mirrored in the performance compared to RT-PCR positive samples with the ELISA having a sensitivity of 78.3% compared to 68.9% for the rapid.

**Table 4:** Human serum panel from Malaysian study site (n = 293).

Days post-onset of illness (DOI)	RT-PCR				Virus Isolation	IgM Capture ELISA	Negative	Total each DOI
	DENV-1	DENV-2	DENV-3	DENV-4				
1	1	0	2	4	4	0	0	8
2	7	2	3	5	4	2	0	20
3	28	4	8	1	13	24	0	45
4	35	5	3	4	7	32	0	60
5	22	7	4	2	1	42	0	50
6	2	3	2	0	1	28	0	29
7	0	0	1	0	0	21	0	21
8	6	0	0	0	0	9	0	9
9	0	0	0	0	0	2	0	2
10	0	0	0	0	0	1	0	1
11	0	0	0	0	0	3	0	3
12	0	0	0	0	0	7	0	7
13	0	0	0	0	0	6	0	6
14	0	0	0	0	0	1	0	1
15	0	0	0	0	0	1	0	1
UNKNOWN*	0	0	0	0	0	0	30	30
Total	101	21	23	16	30	179	30	

*No information was available for day post-onset of illness that negative specimens were collected.

A small number of dengue negative samples that were characterised as positive for other viral aetiologies were included as specificity controls. Of the 30 dengue negative samples tested, the NS1 rapid test had a specificity of 96.7% (95% CI: 82.8 – 99.9) with one false positive result observed. The problematic sample was collected from a patient infected with Cytomegalovirus (CMV).

**Figure 3:**
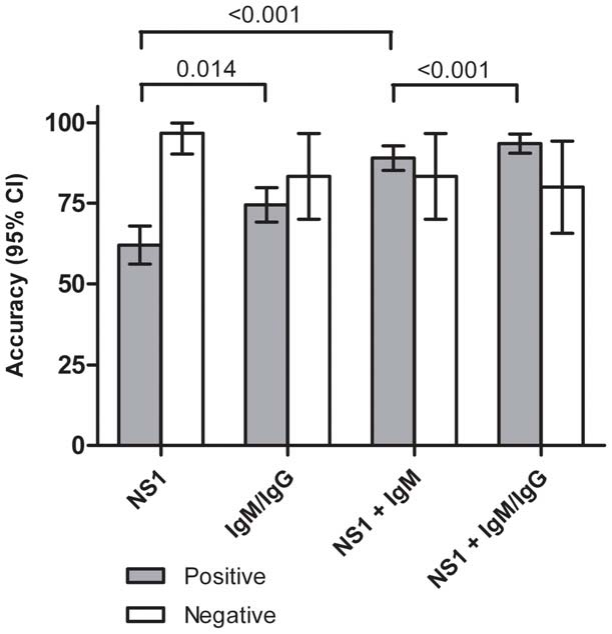
Accuracy of dengue rapid tests at the Malaysian study site. Diagnosis was confirmed using a combination of HI, virus isolation, RT-PCR and IgM ELISA (n = 293). Sensitivity (%) between groups was compared using Fisher's exact test.

The Malaysia study assessed the clinical utility of using the Dengue Early Rapid alone and in combination with the Dengue Duo Cassette (Figure 3). As individual tests the sensitivity of the Dengue Early Rapid and Dengue Duo Cassette was 62.0% and 72.5% respectively. The combination of NS1 detection with anti-IgM detection resulted in a significant (P<0.001) increase in sensitivity to 89.0% (95% CI: 85.2 – 92.8) compared to detection of NS1 alone (Figure 3), with the caveat that a single IgM positive test provides a presumptive, not confirmatory, diagnosis. Sensitivity was further improved to 93.0% by combining all three dengue markers (NS1, anti-IgM and anti-IgG); however the higher sensitivity was offset by a slight loss of specificity.

**Figure 4:**
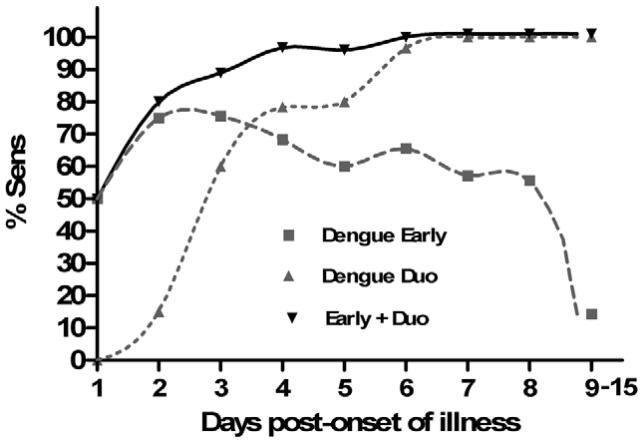
Clinical sensitivity of rapid tests for acute dengue over the course of illness. Data was obtained from laboratory-confirmed cases of dengue at the Malaysian study site (n = 263).

The sensitivity of the antigen and antibody rapid tests at each day post-onset of illness highlighted the clear difference between the windows of detection between the two tests (Figure 4). As expected, the antigen rapid test had greater sensitivity early in the course of infection, with a peak sensitivity of 75% at days 2 and 3. NS1 sensitivity decreased from day 3 onwards and coincided with the rise in detection of antibodies. From day 6 the IgM/IgG rapid test showed >95% sensitivity.

## Discussion

In this study we report on the performance of a new commercial rapid test for dengue NS1, evaluated at two Southeast Asian study sites. The test was developed using a combination of monoclonal antibodies capable of detecting all four serotypes of the antigen at low ng/mL concentrations. This promising analytical sensitivity translated to good diagnostic performance in a clinical setting with sensitivity and specificity that was comparable to other commercially available NS1 rapid tests evaluated in the same endemic region [24]. Overall, we found that optimal diagnostic sensitivity for acute dengue was achieved when NS1 testing was combined with IgM and IgG detection, with a significant increase in sensitivity compared to the outcome from using each individual marker.

Despite NS1 becoming an established and very useful diagnostic marker for early detection of acute dengue infection [11], [18], [23], [25], differences in the kinetics of its disappearance from the sera of primary versus secondary infections means that caution should always be exercised in interpreting findings based on NS1 detection alone. With the rapid rise of an anamnestic antibody response to shared epitopes in secondary infections, NS1 levels may be quickly masked by circulating antibody and/or cleared from circulation. Hence, we and others [24], [26] have shown that the most effective diagnostic application of NS1 detection is when it is combined with antibody detection. Together, they provide a broader window of detection than was possible with traditional antibody-based serological assays. Direct detection of viraemia by RT-PCR shares a similar window of detection to the presence of NS1. However it has become apparent that there is not a precise correlation between the two markers, with NS1 often detected in the absence of RNA and vice versa [10], [27]–[29]. Although originally viewed as a surrogate marker for viraemia, circulating levels of NS1 are more likely a measure of infected cell mass. Degradation and/or clearance from circulation of these two infected cell products are also likely to vary [17].

Data from this study supports a growing body of evidence that a combination of NS1 and IgM/IgG detection provides increased diagnostic sensitivity. As individual tests the sensitivity of the Dengue Early Rapid and Dengue Duo Cassette was 62% and 72.5%, respectively, but when used together the sensitivity was increased to 93%. The two tests have complementary windows of detection and we observed a gradual improvement in sensitivity from as early as day 2 post-onset of illness with the combined antigen and antibody approach. Tricou et al.[24] recently compared the performance of two other commercial NS1 rapid tests and identified that combined NS1 and IgM/IgG testing increased the overall test sensitivity without reducing specificity. However unlike the Tricou study we observed a reduced specificity with the combined Panbio tests, with the majority of false positives arising from the IgM/IgG rapid assay. However, considering the specificity of our combined antigen and antibody testing approach was determined based on a very small number (n = 30) of potentially cross-reactive samples and that there have been contrasting reports of dengue IgM and IgG ICTs having either very good specificity of >95% [30] or more moderate specificity [31], [32], an expanded study with a larger population of endemic negative samples will be required to more accurately assess the overall impact on specificity by combining both tests.

In both the Vietnam and Malaysia evaluations, the dengue positive population was heavily skewed towards DENV-1 infection with much lower proportions of DENV-2, DENV-3 and DENV-4 infections. Although an equivalent analytical sensitivity has been demonstrated for all four serotypes, these theoretical limits of detection were established using recombinant proteins developed using gene sequences from East Timor, New Guinea and Puerto Rican clinical isolates and may not account for subtle variations in linear or conformational epitopes between strains. Additional clinical data will be needed from populations in which DENV-2, DENV-3 and DENV-4 infections are predominant in order to validate the utility of the test during outbreaks of each serotype. Demonstrating good clinical sensitivity for DENV-2 infections will be relevant considering reports that the concentration of NS1 in serum may be lower relative to DENV-1 and DENV-3 infections [11], [17]. There have also been reports of reduced sensitivity towards DENV-3 with other rapid NS1 tests [33], [34]. It will also be important to validate the performance of the combined dengue NS1 and IgM/IgG testing in other dengue endemic regions such as the Americas, Pacific Islands, India and Africa.

The ongoing challenge for dengue serological assays surrounds the need for sensitivity towards all four related, but antigenically distinct, serotypes whilst at the same time limiting unwanted cross-reactivity to other members of the flaviviridae family that co-circulate in dengue endemic regions, such as Japanese Encephalitis, and Yellow Fever. There is scope to improve on assay sensitivity with the development of improved recombinant antigens and capture antibodies with faster on-rates, which are in progress. False positivity can occur as a result of cross-reactive antibodies from a previous flavivirus infection and auto-immune complexes [2]. NS1 has been recognised as a highly specific serological marker and provides an expanded window of detection when included with traditional antibody detection. Tricou et al [24] and Hang et al [11] have observed 100% specificity using the BioRad® NS1 rapid test in clinical evaluations in Vietnam and in these clinical studies we have observed >95% specificity with the Dengue Early Rapid test. The very high specificity of these tests combined with their early window of detection and ease of use make them an ideal candidate for use as a surveillance tool.

In conclusion, we have demonstrated that the Panbio® Dengue Early Rapid is both a sensitive and specific rapid test for dengue NS1. When used in combination with an IgM/IgG rapid test a significant increase in sensitivity for acute dengue was observed with the added benefit of an extended window of detection. The use of rapid ICTs for dengue has become increasingly popular, particularly in developing countries, due to their simplicity, affordability and their suitability for use at or near the point of care. The availability of NS1 to complement antibody detection further enhances the clinical utility of this platform. At both study sites, the predominant infecting serotype was DENV-1 and ongoing clinical evaluation of this combined antigen and antibody testing protocol will be required during outbreaks with other serotypes as well as in other endemic regions outside of Southeast Asia. The focus of this investigation was improved sensitivity of dengue diagnosis but there is also a need to more comprehensively evaluate the effect of this combined approach on specificity and ultimately positive predictability.

## Supporting Information

Figure S1 **Dengue laboratory testing algorithms used at the Vietnam and Malaysia study sites.**
(TIF)
