# Supplementary figures and images for: The Diagnostic Sensitivity of Dengue Rapid Test Assays Is Significantly Enhanced by Using a Combined Antigen and Antibody Testing Approach
Source: PLoS Negl Trop Dis. 2011 Jun 21;5(6):e1199. doi: 10.1371/journal.pntd.0001199 (PMC3119643; doi:10.1371/journal.pntd.0001199)

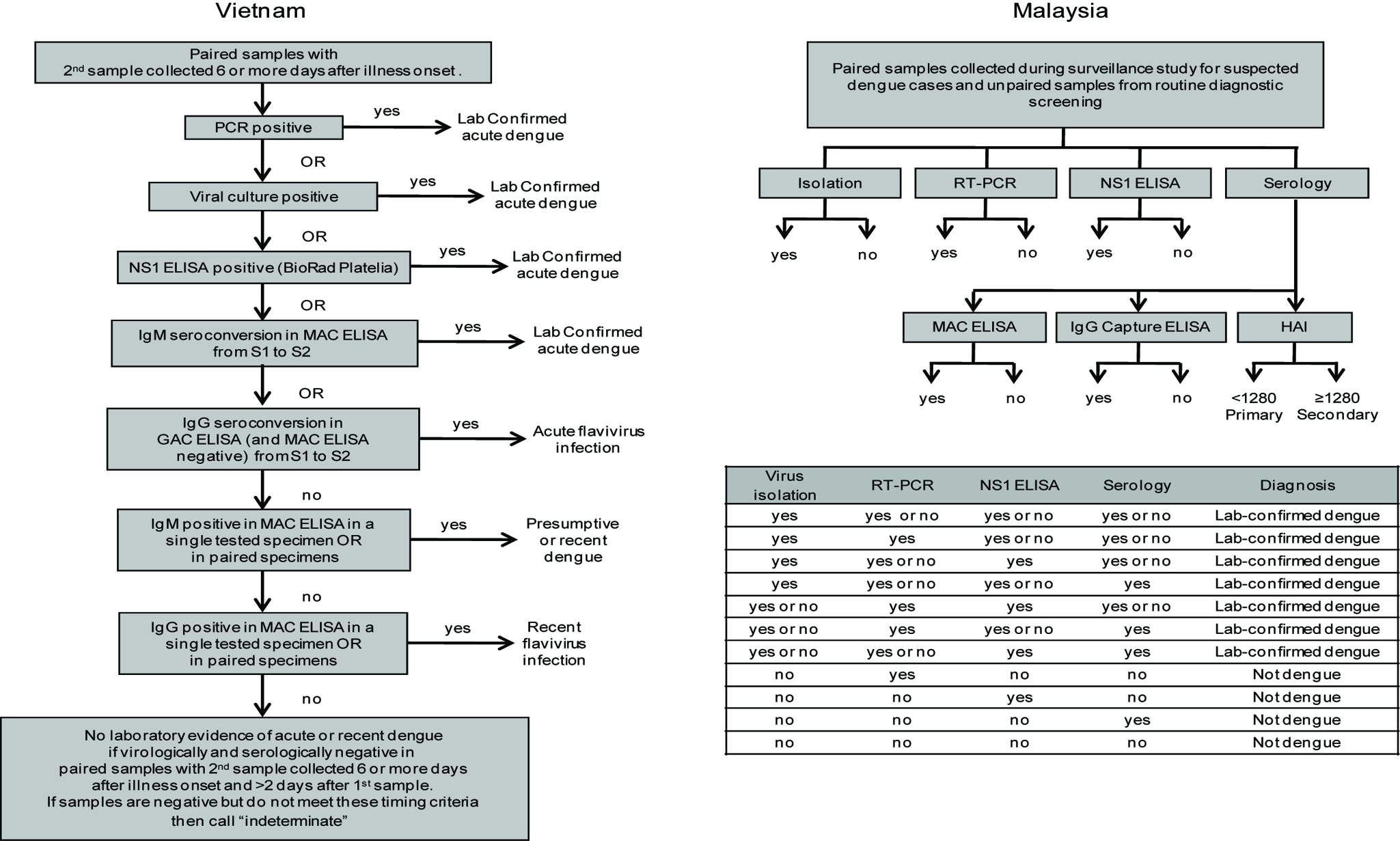

Supplement: Figure S1 — Dengue laboratory testing algorithms used at the Vietnam and Malaysia study sites. (TIF) [file pntd.0001199.s001.tif]
